# Awareness and attitude of the general public toward HIV AIDS in coastal South India - a community based crossectional study

**DOI:** 10.1186/1742-4690-9-S1-P105

**Published:** 2012-05-25

**Authors:** B Reshmi, B Unnikrishnan, P Mithra, T Rekha

**Affiliations:** 1 Manipal College of Allied Health Sciences, Manipal, India

## Background

Acquired Immunodeficiency Syndrome (AIDS) is one of the most dreaded entities that modern medicine has ever had to tackle. Adult HIV prevalence in India is approximately 0.36%. HIV-related stigma and discrimination remains an enormous barrier to effectively fighting the HIV and AIDS epidemic, There are several reasons for the stigma toward PLWHA among the general population, one of them could be inaccurate information about the transmission of HIV; creating irrational behavior and misperceptions of personal risks.

## Objective

To assess the awareness and attitude of the general public toward people living with HIV/AIDS (PLWHA) in Mangalore, a city in Coastal Karnataka.

## Methods

The study population included 630 individuals aged 18 years and above. The information was collected using a semi structured pre-tested questionnaire. Statistical package SPSS version 11.5 was used, Chi-square test was conducted and P< 0.05 was considered as statistically significant. Results: About one-third of the study population thought that one could get infected by merely touching an HIV positive individual. Approximately 45% stated that they would dismiss their maid on finding out her HIV positive status. About 54% were willing to undergo the HIV test. The respondents with less than secondary school education had a discriminatory attitude toward HIV positive people, with regard to them deserving to suffer, dismissing a HIV positive maid, hesitating to sit next to a HIV positive person in the bus, divorcing the infected spouse, and willingness to get tested for HIV, which was found to be statistically significant.

## Conclusion

Stigma among the general public was mostly due to fear of contracting the illness. Stigma does exist to significant degrees among the educated people, which was suggested by about 45% of the participants being willing to undergo the HIV test.

